# Supplementary material for: Identification of Freezing-Responsive microRNAs and Their Targets in Chinese Jujube by Small RNA and Degradome Sequencing
Source: Int J Mol Sci. 2026 Sep 4;27(17):7912. doi: 10.3390/ijms27177912 (PMC13566890; doi:10.3390/ijms27177912)
Supplement: Supplementary file 1 [file ijms-27-07912-s001.zip › Supplementary Material S2/novel_miR_31.pdf]

Provisional ID	:	novel_miR_31
Score total	:	0.4
Score for star read(s)	:	-1.3
Score for read counts	:	0
Score for mfe	:	0.1
Score for randfold	:	1.6
Score for cons. seed	:	
Total read count	:	55
Mature read count	:	53
Loop read count	:	0
Star read count	:	2

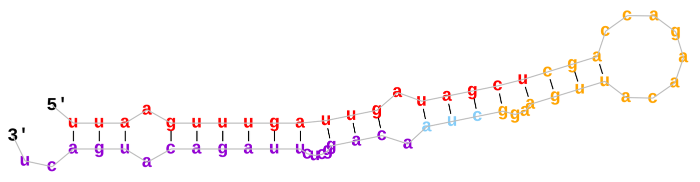

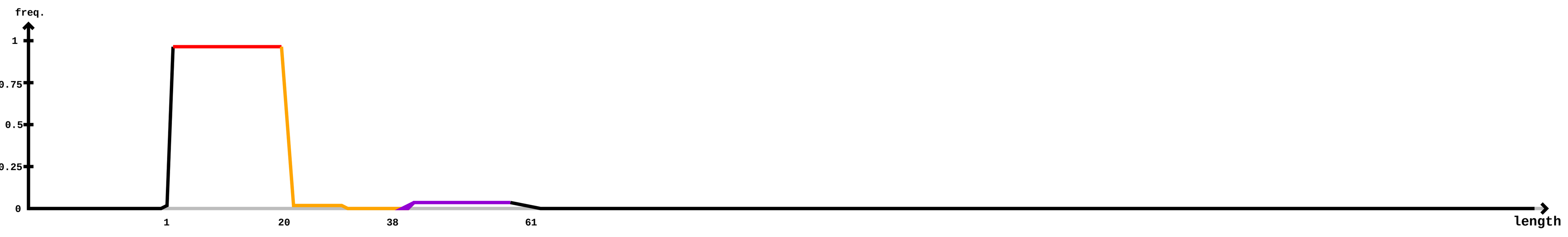

Mature Star

```
5'- aaaauuaguuucucuacucuccuuaaguuugauugauagcucgaccagaacauugaaggcuaacaggcucuuagacaugacuggagauagagaugcauucucaauaaugguugacagcuuaacaaacucaagcaagucuccugacaggucagguaagacagcauauaaaagcaucugcaauugccaagugugagccaauagacaauaaacuuuaaggcauuaaucuaaguuuacuuauaagagcucacac -3' obs
    aaaauuaguuucucuacucuccuuaaguuugauugauagcucgaccagaacauugaaggcuaacaggcucuuagacaugacuggagauagagaugcauucucaauaaugguugacagcuuaacaaacucaagcaagucuccugacaggucagguaagacagcauauaaaagcaucugcaauugccaagugugagccaauagacaauaaacuuuaaggcauuaaucuaaguuuacuuauaagagcucacac exp
    .......((.((((((.((((((((.(((((((((.((((((((........)))))..))))).)))....))))))).))))..))))))))))).))....(((((....)))))..((((.........))))..(((((((((....)))))..))))..........(((........)))...((((((((.........(((((((..(((.....))))))))))).........)))))))) reads mm sample
    .....................cuuaaguuugauugauagcucgaccagaac.............................................................................................................................................................................................. 1 0 LT-1
    ......................uuaaguuugauugauagcu............................................................................................................................................................................................................ 20 0 LCK-2
    ......................uuCaguuugauugauagcu............................................................................................................................................................................................................ 1 1 LCK-2
    ......................uuaaguuugauugGuagcu............................................................................................................................................................................................................ 1 1 LCK-1
    ......................uuaaguuugauugauagcu............................................................................................................................................................................................................ 31 0 LCK-1
    ..............................................................acaggcucuuagacaugacu................................................................................................................................................................... 2 0 LT-3
```
